# Breaking the Hydrophobicity of the MscL Pore: Insights into a Charge-Induced Gating Mechanism

**DOI:** 10.1371/journal.pone.0120196

**Published:** 2015-03-31

**Authors:** Balasubramanian Chandramouli, Danilo Di Maio, Giordano Mancini, Vincenzo Barone, Giuseppe Brancato

**Affiliations:** 1 Scuola Normale Superiore, Piazza dei Cavalieri 7, I-56126, Pisa, Italy; 2 Istituto Nazionale di Fisica Nucleare (INFN), sezione di Pisa, Largo Bruno Pontecorvo 3, 56127, Pisa, Italy; Zhejiang University, CHINA

## Abstract

The mechanosensitive channel of large conductance (MscL) is a protein that responds to membrane tension by opening a transient pore during osmotic downshock. Due to its large pore size and functional reconstitution into lipid membranes, MscL has been proposed as a promising artificial nanovalve suitable for biotechnological applications. For example, site-specific mutations and tailored chemical modifications have shown how MscL channel gating can be triggered in the absence of tension by introducing charged residues at the hydrophobic pore level. Recently, engineered MscL proteins responsive to stimuli like pH or light have been reported. Inspired by experiments, we present a thorough computational study aiming at describing, with atomistic detail, the artificial gating mechanism and the molecular transport properties of a light-actuated bacterial MscL channel, in which a charge-induced gating mechanism has been enabled through the selective cleavage of photo-sensitive alkylating agents. Properties such as structural transitions, pore dimension, ion flux and selectivity have been carefully analyzed. Besides, the effects of charge on alternative sites of the channel with respect to those already reported have been addressed. Overall, our results provide useful molecular insights into the structural events accompanying the engineered MscL channel gating and the interplay of electrostatic effects, channel opening and permeation properties. In addition, we describe how the experimentally observed ionic current in a single-subunit charged MscL mutant is obtained through a hydrophobicity breaking mechanism involving an asymmetric inter-subunit motion.

## Introduction

**Fig 1:**
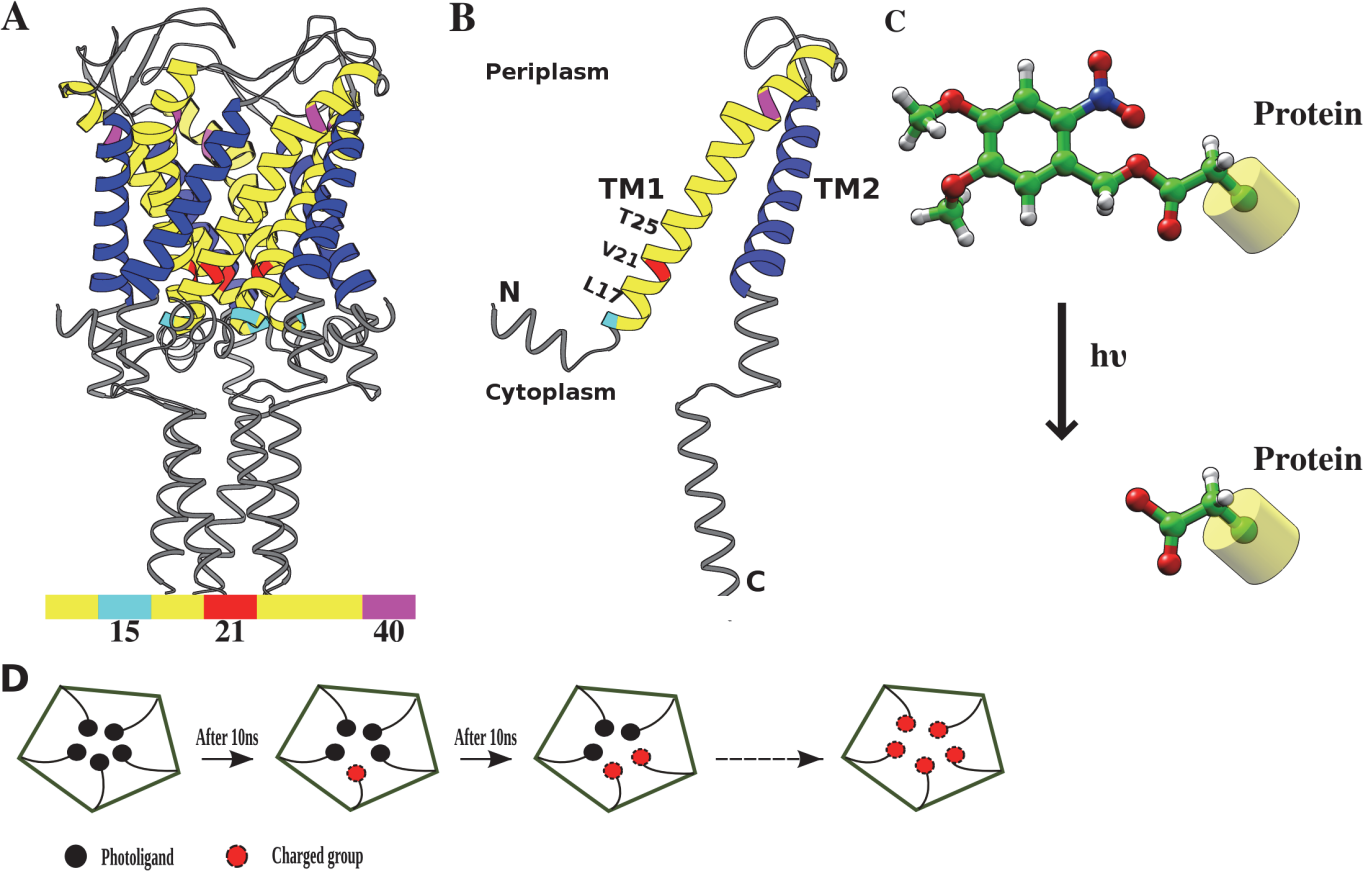
Structure of Tb-MscL and photo-sensitive compound. (A) X-ray crystallographic structure of Tb-MscL (PDB ID: 2OAR) with the transmembrane helices TM1 and TM2 colored in yellow and blue, respectively. Locations of residues lining the inner helix (TM1) are indicated. (B) Single subunit of Tb-MscL. (C) Structure of photo-sensitive compound that releases the charged group upon light irradiation. (D) Schematic view of the step-by-step substitution process followed in the MD simulation protocol.

Mechanosensitive channels are integral proteins located on the bacterial cytoplasmic membrane, whose function is that of a natural pressure valve preventing cell lysis in case of hypoosmotic stress.[1,2] Among the members of this protein family, the mechanosensitive channel of large conductance (MscL) from *E*.*coli* (Eco-MscL) is the best-known protein channel.[3,4] In response to increased membrane tension, Eco-MscL can form a large pore with a diameter of up to ~30 Ang. In the open state, the MscL pore has a non-selective conductance of 3 nS and it allows the passage of large hydrated solutes and small proteins down the concentration gradient. The structure of a MscL homologue from Mycobacterium tuberculosis (Tb-MscL), resolved by X-ray crystallography, has shown a symmetric homo-pentamer,[5,6] in which each subunit consists of a short N-terminal helix, two membrane spanning helices (named TM1 and TM2) connected by a periplasmic loop and a cytoplasmic helix (Fig. 1A). The five TM1 helices form the pore lumen that is characterized by a hydrophobic stretch of residues (I14-V21) creating a constriction at the cytoplasmic side (Fig. 1B), while the TM2 helices form contacts with the surrounding lipid bilayer. The X-ray structure of Tb-MscL represents the closed-state configuration of the protein channel with a radius of ~2 Ang along the narrowest region of the pore and provides the framework with which extensive mutagenic and functional data have been interpreted.

Mutagenesis experiments in Eco-MscL identified residues L19-V23 to be critical for channel gating;[7] the corresponding residues (L17-V21) in Tb-MscL are located in the narrowest part of the pore (Fig. 1B). The selective mutation of these residues increased the feasibility of channel opening, leading to slow/no-growth gain of function (GOF) phenotypes.[8] Further, systematic substitutions of G22 in Eco-MscL by all other amino acids showed that hydrophilic residues at this position resulted in channel gating at reduced tension with respect to the wild-type, while hydrophobic residues made the channel harder to open.[9] Several studies showed that the introduction of charged residues at the same position led to an activated channel in the absence of any applied tension (e.g., mutating the hydrophobic pore residues to Cysteine and attaching the charged methanethiosulfonate (MTS) reagents).[10–12] These findings, together with the ability of MscL to form a large pore and assemble into a fully functional protein when reconstituted into synthetic lipids, led to conceive MscL as an ideal candidate to function as a triggered nanovalve. Indeed, recently engineered MscL channels have been designed to respond to stimuli like pH or light in applications tailored to molecular sensing or drug delivery.[13–15] The light controlled channel gating is particularly interesting as it provides a finer temporal control and does not require any change in the system environment, as reported recently by Koçer *et al*. [15]: in this case, a light-triggered gating has been artificially introduced by a photo-sensitive functional group alkylated with a purposely mutated Cysteine in the pore interior (Fig. 1C). Hence, it has been shown how the application of light has induced a spontaneous channel opening, in the absence of membrane tension, according to a designed charge-repulsion based trigger. On the other hand, in more recent studies by Koçer and coworkers[16,17] it has been shown that MscL channel gating can be also triggered by just one charged group in a single subunit, therefore following a charge-induced hydrophobic breaking mechanism. Overall, these experiments have demonstrated the possibility to i) easily manipulate MscL pore size, ii) control the flux of charged compounds and iii) deliver bioactive molecules into the cell, thus showing the versatile use of MscL as a controllable nanoscopic valve.

In light of such remarkable applications, several aspects of the charge-induced gating mechanism deserve further investigation, among which i) the scale of protein conformational changes upon charge introduction, ii) the extent of the channel structural expansion and iii) the selectivity and geometric limitations of molecular transport across the channel. Such details demand an atomistic level understanding of the interplay between the induced electrostatic effects and the protein structural rearrangements. In this regard, molecular dynamics (MD) simulations can be profitably used to provide structural characteristics and dynamical features of protein channels, while retaining explicitly the environmental effects mainly due to the lipid bilayer and the solvent. Previous computational studies have focused on the gating conformation transitions of the wild-type MscL protein [18–21], the mutation effects in the pore hydrophobic region [22] and the lipid composition effects [23,24]. In the present study, we have performed atomistic MD simulations to investigate the effects of charge incorporation on the hydrophobic pore of Tb-MscL. In analogy with the experimental setup of ref. [15], five photosensitive ligands, one for each protein chain, have been incorporated into a constricted site of a Tb-MscL mutant (V21C). Then, each ligand was sequentially replaced by a charged acetate group (Fig. 1D) and the resulting systems simulated independently. The structural features of the engineered models have been compared with a wild-type model of Tb-MscL. Further, the effects of charge incorporation at two alternative sites has also been examined, considering the L17C and T25C mutants. In addition, we considered a different Tb-MscL model, in which a charged group was attached to only one subunit, mimicking the charge-induced hydrophobic breaking process previously reported.[16,17] The obtained results report new molecular details on channel structural opening, ion permeation properties, asymmetric subunit motions, and site sensitivity towards charge perturbations, therefore providing further insights into MscL charge-induced gating.

## Materials and Methods

### Model generation and simulation details

In ref. [15], light-actuation of MscL has been achieved attaching a photo-sensitive compound (6-nitroveratryl alcohol), which splits into 6-nitrosoveratyl aldehyde and a free acid upon light irradiation, to a G22C mutant of Eco-MscL through a Cysteine-selective alkylating reagent. UV photolysis resulted in Cysteine-bound acetates (hereafter referred to as the charged group) in the pore lumen (Fig. 1C) and consequent channel gating. Here, we attached the same photo-active ligand in all subunits at residue 21, the most constricted site of the Tb-MscL. The starting structure of the MscL channel was embedded into a homogeneous lipid bilayer of DOPC(1,2-dioleoyl-*sn*-glycero-3-phosphocholine) spanning ~100 Ang along the lateral dimensions (x,y) using the CHARMM-GUI server,[25] followed by solvation with TIP3P water, extending up to 30 Ang from the solute in the axial dimension (z-axis) using VMD.[26] Since the constricted site is extremely narrow to fit the ligands without steric conflicts, the channel was slowly expanded through multiple short simulations by placing an uncharged Lennard-Jones (LJ) atom at residue C21 centroid and increasing its LJ radius parameter. Once sufficient space was created, the ligands were attached to C21 in all five subunits. K^+^ and Cl^-^ were added to the system, setting the ion concentration to about 1 M. The engineered model with five ligands (hereafter referred to as 5L) was then minimized to remove steric clashes, slowly heated up from 100 K to 300 K in about 1 ns simulation and then equilibrated for 10 ns in a NPT ensemble. The production run was performed in a NVT ensemble, applying a homogeneous external electric field (E_*z*_) along the z-axis (L_z_) perpendicular to the membrane proportional to a defined voltage (V = 1 Volt; E_*z*_ = -V/L_z_). Different engineered models were then generated by a sequential replacement (every 10 ns) of each ligand by the charged group (Fig. 1D). The starting models were equilibrated for 6–8ns, followed by extended production runs in the presence of electric field. To verify the charge effects on the pore, a model representing the wild-type (WT) protein was simulated using a snapshot of NL model (obtained after 50 ns) as the starting configuration and replacing the charged group in all subunits with Valine. To investigate the charge activation of a single subunit in a different MscL model, we extracted an equilibrated configuration of the WT model and mutated residue V21 to Cysteine in a single subunit, to which the same charged group was attached. Other simulations involving alternative sites were initiated from the same configuration of the WT model, after mutating the corresponding residues to Cysteine (namely, L17C and T25C) and attaching the corresponding charged group in all subunits. A summary of the simulated systems is reported in Table 1.

**Table 1:** Summary and description of the simulations.

Models	Description (charge^a^)	Time (ns)
5L	5 Ligands at position 21 (0e)	30
4L	4 Ligands, (-1e)	30
3L	3 Ligands, (-2e)	30
2L	2 Ligands, (-3e)	30
1L	1 Ligand, (-4e)	50
NL	No Ligand, (-5e)	100
WT	Wild type (0e)	50
WT_1e	WT with charge in a single chain (-1e)	80
17M	5 charges at position 17 (-5e)	50
25M	5 charges at position 25 (-5e)	50

^a^Total charge at the functionalized site.

All simulations were performed enforcing periodic boundary conditions using NAMD (ver. 2.9) [27] and the CHARMM force field for the protein (v.27) and lipids (v.36) [28–30]. The ligand atomic charges were obtained via fitting with a quantum-mechanically derived electrostatic potential issuing from B3LYP/6-31G* calculations using the Gaussian package,[31] according to the restrained electrostatic potential (RESP) procedure using the RED software tools [32]. The parameters for the ligand and the acetate groups were adopted from the CHARMM CGEN small molecule force field [33]. All covalent bonds with hydrogen atoms were kept rigid using the SHAKE algorithm, allowing the use of a 2 fs time step for the integration of the equations of motion. A cutoff radius of 12 Ang was used for non-bonded interactions, applying a smoothing function beyond a distance of 10 Ang. The long-range electrostatic interactions were evaluated with the Particle Mesh Ewald (PME) [34] method, whereas a Langevin dynamics was used to maintain the system temperature at 300 K. During equilibration, the pressure was kept at 1 atm using the Langevin piston method with a piston period, damping coefficient and piston temperature of 100fs, 50fs and 300K, respectively.

### MD trajectory analyses

Root mean square deviations (RMSD) were computed performing a least-square fitting to a reference structure, either the starting one or the X-ray crystallographic structure. The pore radius profile was obtained using the HOLE program.[35] The ion flux was estimated by calculating the number of ions entered from one side and exited through the opposite side of the channel (considering the TM domain only) with an in-house code written using MDANALYSIS.[36] The analysis was performed omitting the first 10 ns of the MD trajectory during which the models undergo structural rearrangements. The pore area was approximated by the area of a regular pentagon by setting as its edge length the average distance between C^α^ atoms in adjacent subunits. Water and ions around a permeating K^+^ were estimated from the average number of corresponding species within a predefined cut-off radius, as a function of the z-coordinate. Channel conductance was estimated from the ratio of the average current and applied voltage.[37,38] Inter-subunit contacts were obtained considering the C^α^ atom distances. Trajectory visualization was performed using the Caffeine software.[39] Figures and plots were generated using UCSF-CHIMERA and MATPLOTLIB software.[40,41] Unless stated otherwise, the axial positions in all plots are indicated with respect to the origin fixed at the C^α^ geometric centroid of residue 21.

### Free energy calculations

A computational approach similar to that reported in previous studies [42,43] was employed to estimate the potential of mean force (PMF) governing K^+^ translocation through the channel using the adaptive biasing force methodology (ABF) implemented in the NAMD package.[44,45] In short, the adopted method (see Ref. [45] for a more detailed discussion) relies on the selection of a predefined coordinate, which, in the present work, corresponds to the z-coordinate (normal to the membrane) of a testing ion. The average force acting on the ion along such a coordinate is estimated from a large number of instantaneous force evaluations. The average force is collected in bins and updated during the simulation. An adaptive biasing potential, equivalent and opposite to the average force is applied after a defined number of samples in each bin to overcome any energy barrier that prevents ion translocation. The PMF was evaluated along the z-coordinate (channel axis), considering a region that extends up to ~10 Ang on either side of the functionalized site. The simulations were performed in 10 separate windows of 2 Ang length for about 11 ns. For each window, the starting coordinates were extracted from the production runs described above. Excess ions were removed, retaining only those required for charge neutralization including a single K^+^ ion in the pore. The ions in the bulk solution were restrained to obtain the single-ion PMF profile.

## Results

### Structural rearrangements of the engineered MscL channels

The Tb-MscL protein is a relatively small homopentamer channel of 151 residues (17KDa) (Fig. 1A). In particular, each monomer has two transmembrane α-helices, namely TM1 and TM2, where TM1, one of the most conserved motifs of the mechanosensitive channel family, forms the inner core of the channel and is mostly hydrophobic (V^15^DLAVAVVIGTAFTALVTKFTDSIIT^40^). First, we have analyzed and compared the structures of different engineered MscL channels issuing from the sequential replacement of the attached (neutral) photo-sensitive ligands, i.e. 6-nitroveratryl alcohol, by negatively charged acetate groups. It should be noted that in the original experimental work[15] only the MscL channels carrying either the five neutral ligands (5L system) or the corresponding photoproducts (NL system) were presumably detected. Nevertheless, our purpose was to study in some detail the effect of each charge insertion on the MscL channel properties, so as to investigate the virtual realization of a multi-state gating mechanism (a proof-of-principle of a multi-state MscL channel activation was provided in ref. [16], even if the gating mechanism was not controllable by light). For such a purpose, all systems were subjected to an applied voltage (1 Volt) and a 1 M KCl salt concentration.

The stability of the simulated MscL models was assessed by evaluating the structural deviations from starting configurations. RMSD of the backbone atoms, depicted as function of simulation time, have shown a plateau after about 10 ns (S1 Fig.), following an initial increase due to structural relaxation. The distribution of RMSD values over the last 20 ns has shown an average deviation of ~2 Ang (S1 Fig. and S1 Table). The RMSD from the X-ray structure of the whole protein were generally higher (about ~3 Ang), but considerably reduced when the TM or C-terminal domains were separately examined (S1 Table). In summary, all models displayed an apparent convergence towards an equilibrium conformation in a few nanoseconds and a stable structural arrangement afterwards during the simulations. Moreover, the flexibility of the MscL protein was examined by evaluating the backbone structural fluctuations. To this end, the root mean square fluctuations (RMSF) mapped onto the average structures (S2 Fig.) have shown that the most mobile regions are localized mainly on the periplasmic loop of the protein. The smaller fluctuations in the TM region indicated the structural integrity of this region upon ligand or charge incorporation.

### Structural expansion of the channel

**Fig 2:**
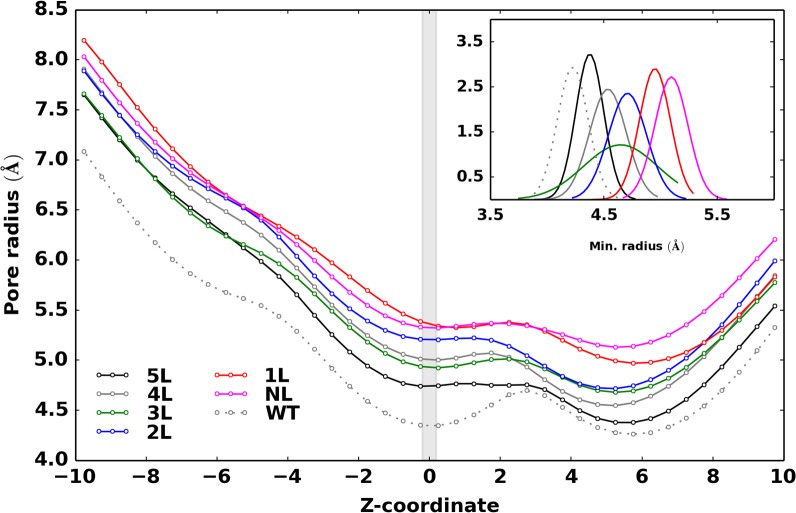
Average pore radius along the channel. Average pore radius along the channel axial position (Z-coordinate) with respect to the mutated site (shaded portion, Z = 0 Ang). Inset plot represents the distribution of minimum pore radius calculated by limiting the analysis to the constricted zone of the pore (residues 17 to 23).

The extent of channel structural expansion in each engineered MscL model was examined by evaluating the average pore radius along the channel axis, considering only the backbone atoms for the sake of comparison (i.e. the steric effects of side chains and ligands were neglected). In Fig. 2, the average pore radius as a function of the axial position is depicted for all models, including the wild-type protein. The pore radius profile has shown a gradual expansion of the channel following the sequential incorporation of additional negative charges into the channel core. A rather similar degree of expansion is observed along the whole TM1 helix, suggesting its tendency to behave as a rigid body. The distribution of the minimum pore radius (see inset of Fig. 2) has provided a monotonic shift (5L<4L<3L<2L<1L<NL) towards larger radius values in going from 5L to NL. Hence, the observed trend in pore radius supported an increasing charge effect, as previously observed on charge-mediated MscL gating.[16] To further estimate the extent of channel opening, we have approximated the area of the pore by evaluating the area of a regular pentagon with edge given by the average distances of adjacent C^α^ atoms (i.e., on proximal subunits) at the same residue along a stretch of aminoacids flanking the functionalized site. Results depicted in S3 Fig., once again indicated a larger expansion of the NL model as compared to the 5L, in line with the pore radius profile (Fig. 2). However, the maximum channel opening was quite smaller than the one reported for membrane tension and charge-induced mechanism (up to ~30 Ang diameter).[46,47] In order to test the overall stability and geometric features of the expanded channel in the NL model, an additional 100 ns MD simulation was performed at high temperature (330 K), but no significant changes have been noticed.

Moreover, it is worth nothing that the WT simulation was initiated from an equilibrated configuration of the NL model after replacing each charged group at the functionalized sites with Valine. Such a hydrophobic substitution led to a fast channel closure (a few nanoseconds) as indicated by the radius profile (Fig. 2). A similar spontaneous channel closure has been observed for MscS channel during unrestrained MD simulations.[48] Note that the pore dimension in the WT model appeared somewhat larger than the corresponding X-ray structure, owing to thermal fluctuations and a more realistic environment (crystal packing forces are generally unleashed in MD simulations of proteins, as observed in a previous MD study on the X-ray structure of Tb-MscL[49]).

### Ion permeation: PMF and translocation mechanism

The ion flux through the functionalized MscL proteins was analyzed by monitoring separately the total number of ions entering into the channel (Entry in Table 2) and the number of ions permeating the channel through opposite vestibules of the pore (Exit in Table 2). Then, a permeation rate has been obtained as the ratio of permeated over entered ions. In all cases, results have been collected from corresponding MD simulations upon application of voltage. Due to the overall small pore dimension and the filtering effect of the negatively charged groups, only K^+^ ions were able to permeate through the engineered MscL channels from the periplasmic side. As reported in Table 2, the permeation statistics for K^+^ ions has shown the occurrence of ion flux only through 1L and NL models. In 1L model, the bulky ligand in the pore largely attenuated the permeation events (~11%), albeit a structural expansion similar to the NL model is observed (Fig. 2). The NL model allowed the highest ion flux (~57%) throughout the entire simulation (a rather similar permeation rate has been also obtained from selected portion of the whole trajectory). In WT simulation, no ion permeation was observed under applied voltage.

**Table 2:** Ion flux (K^+^) through the engineered MscL channels.

Model	Entry (#)	Exit (#)	Permeation (%)
5L	42	0	0
4L	32	0	0
3L	36	0	0
2L	29	0	0
1L	79	9	11
NL	129	77	60
WT_1e	86	33	38

**Fig 3:**
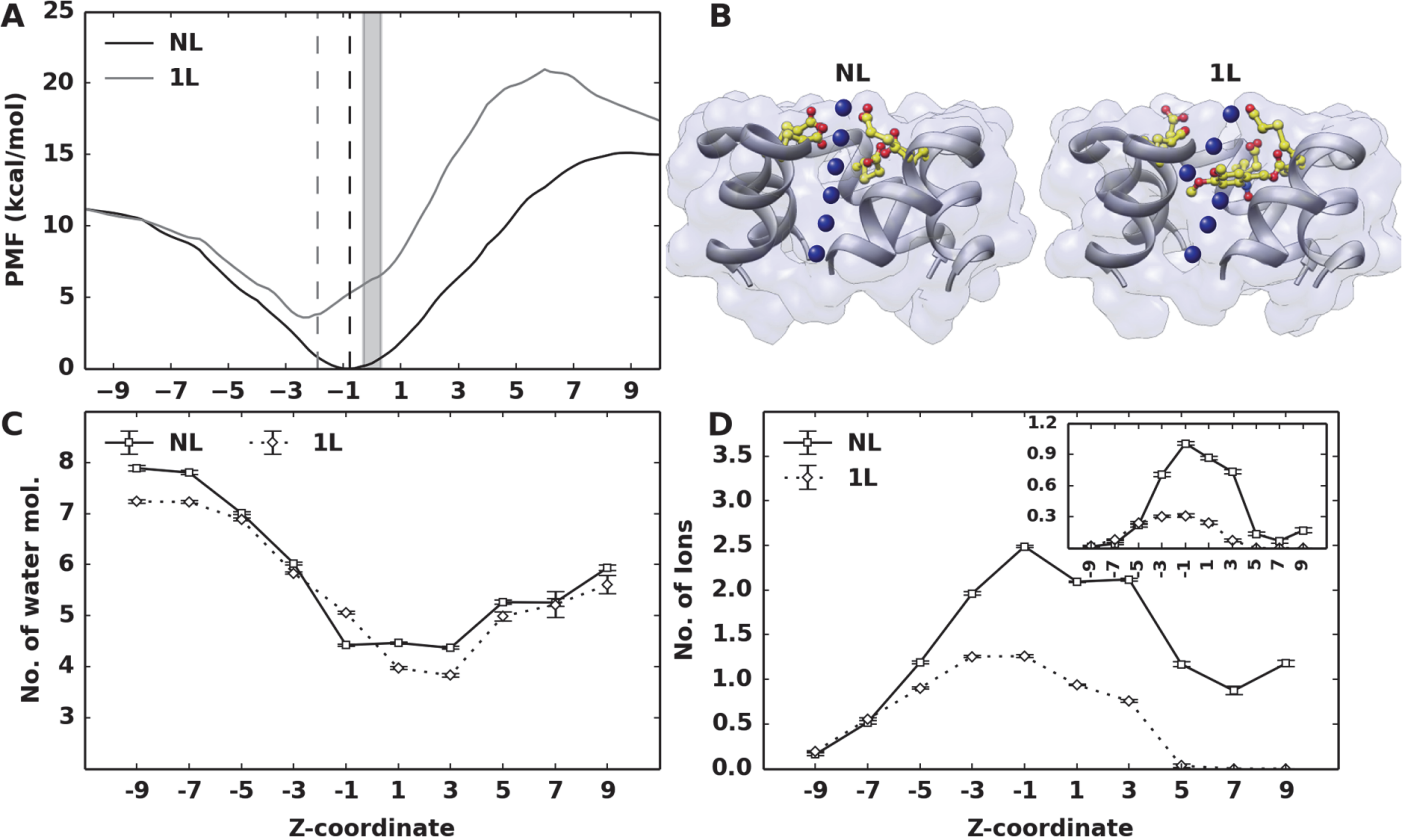
PMF profile, water and ion coordination around the potassium ion. (A) PMF profile as a function of the z-coordinate. The origin was set at the C^α^ geometric centroid of the mutated site. Vertical dashed lines indicate the average location of the acetate group oxygens centroid with respect to the origin. (B) Schematic view of ion exit pathway from the mutated site. The charged group (NL) and the bulky ligand (1L) are shown in yellow. Blue spheres inside the pore represents the K^+^ ion. (C) Average number of water molecules and (D) potassium ions within a radius of 3.5 and 5 Ang, respectively, around the permeating K^+^ ion. Data obtained with a smaller radius (4 Ang) is shown in the inset.

Energetic barrier for K^+^ translocation through 1L and NL models that permit the ion flux was examined by evaluating the single-ion PMF profile along the pore, considering a region that flanks ~10 Ang on either side of the mutated site. The PMF profile (Fig. 3A) reports a downhill in the free energy barrier in both systems as the ion approaches the mutated sites from the periplasmic side (Fig. 3A, shaded region), where it is highly stabilized by the electrostatic attractions of the charged groups. In both cases, an increasing PMF barrier is observed as the ion exited from the cytoplasmic side since this region constitutes a hydrophobic stretch. The higher barrier observed for the 1L model at this stretch can be rationalized as follows: the bulky ligand narrows the channel size and perturbs the local symmetry of the pore lining residues, thereby hindering the linear flux at the exit pathway from the functionalized site (Fig. 3B), providing an additional energetic cost for translocation. On the other hand, the NL model showed a more attractive PMF (~ 2–3 kcal/mol) in the *z* < 0 region as compared to the 1L model, due to the higher total charge at the functionalized site and the absence of any bulky ligand.

**Fig 4:**
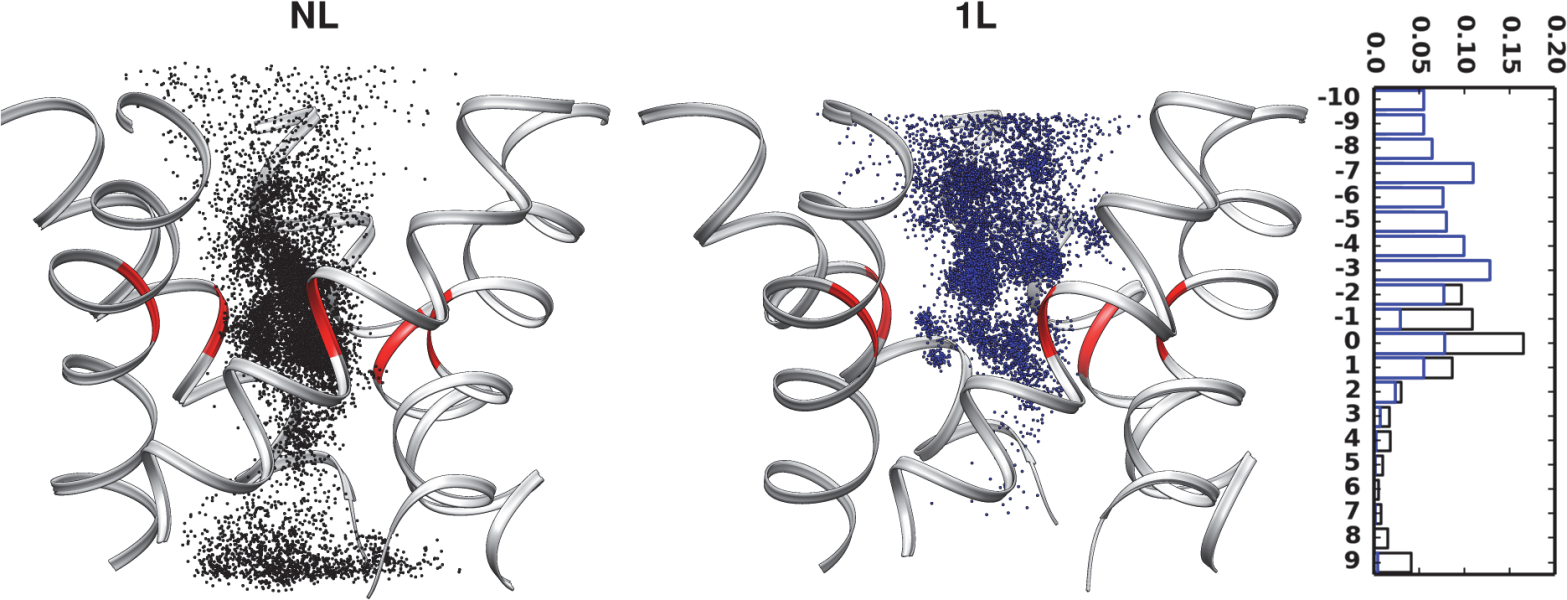
Ion occupancy and histogram of ion counts along the axial positions. Occupancy of K^+^ ion inside the pore obtained by extracting the ions within a distance of 10 Ang from the mutated site along the radial and axial dimensions. Right panel shows the histogram of K^+^ counts along the axial positions for NL (black) and 1L (blue) models.

As evidenced by the single-ion PMF, the electrostatic attractions extended by the ring of charged groups may present an electrostatic trap, restraining the K^+^ ion movement along the pore. To investigate the factors that facilitate the escape from such a strong attractive site, the ion coordination of permeating K^+^ ions has been carefully scrutinized, considering both water and other cations. For each K^+^ ion inside the pore, the average number of water/other K^+^ ions within a spherical region of defined radius has been evaluated. The number of water molecules around the ion has shown to decrease as it approaches the mutated site (Fig. 3C) and such dehydration is similar for both the 1L and NL models. It is reasonable to assume that the loss of water is compensated by the interactions with the charged groups, specifically the oxygen atoms. At variance, the average number of surrounding cations has increased around the mutated site (Fig. 3D). The NL model has shown an average number of up to ~2.5 cations within a sphere of 5 Ang radius (~1 cations within 4 Ang radius, see inset in Fig. 3D). The surrounding cations can give a significant contribution to the permeating ion by counterbalancing the attractive forces of the negatively charged ring, thus aiding the ion to escape from the electrostatic trap described above. In 1L model, the bulky ligand restricts the available pore size and consequently the number of surrounding cations (~1 cations within 5 Ang radius). This result is also confirmed by the average number of K^+^ ions along the pore (see the histogram of K^+^ counts along the axial positions in Fig. 4). The lower cation density at the level of the functionalized site in the 1L model is expected to provide a less effective electrostatic counterbalance, as required for a rapid escape from the highly charged site of the pore. Such a result, in turn, may explain the lower permeation rate observed in the 1L model with respect to the NL model (Table 2).

### Alternative functionalization sites

**Fig 5:**
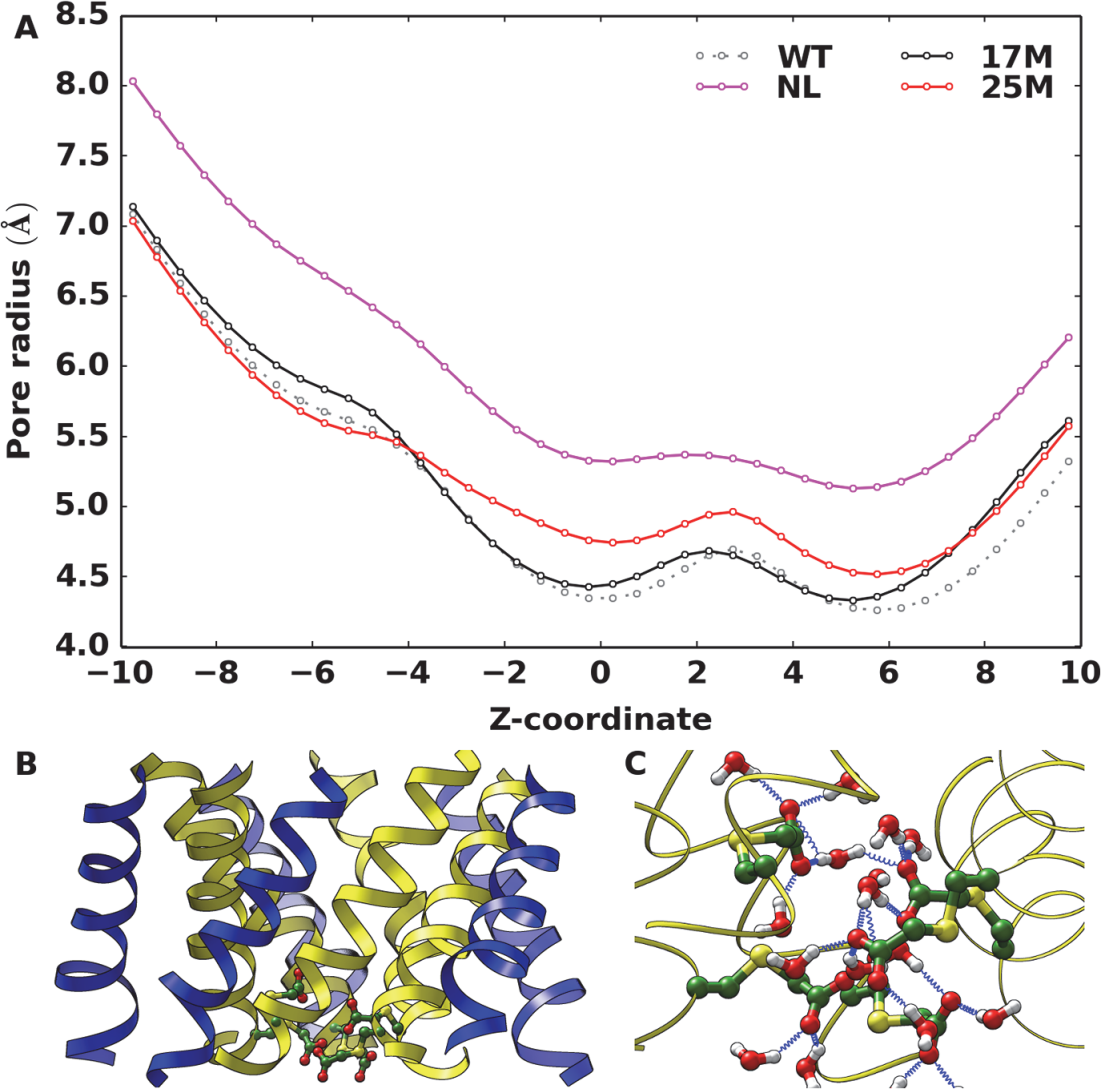
Average pore radius and protein-water interaction at cytoplasmic entrance. (A) Average pore radius along the axial position. (B) Representative snapshot of the 17M model with charged groups shown in green. (C) Interaction of charged groups with water molecules within a distance of 3 Ang (17M model). Protein-water hydrogen bonds are shown as blue springs.

The TM1 helix in Tb-MscL contains other two hydrophobic residues (L17, T25) similarly exposed to the pore with respect to V21, and located on either side of V21. We have investigated the effect of charge incorporation at these alternative sites (Table 1), performing additional simulations of corresponding fully charged systems (i.e., after introduction of the charged group in all five subunits). To be specific, we have generated two additional MscL protein models by attaching the same functional group, i.e. a charged acetate group, at either position 17 or 25 and compared the results with the original engineered protein (NL system). The pore radius profile along the channel axis, as issuing from both models, has shown no appreciable expansion as compared to the result obtained for position 21 (Fig. 5). Overall, the tested models displayed a pore radius similar to the WT protein, indicating the absence of any significant structural transitions induced by the charges. Note that the location of T25 already provides sufficient space to accommodate the charged groups without demanding for any additional structural rearrangements. As a matter of fact, the same pore size has been observed at position 21 and 25 in both corresponding engineered proteins, namely NL and 25M models, with the difference that in the former case a channel expansion has occurred that allows the coordination of K^+^ ions at the pore center. On contrary, residue L17, being located at the narrower entrance at the cytoplasmic edge is expected to be sensitive to charge perturbations (Fig. 5B). However, visual inspection revealed that the charged groups inserted at this position reorient themselves to make stronger interactions with the water molecules that fill the space between the transmembrane and the cytoplasmic domain (Fig. 5C). The interaction with water molecules can effectively shield the charge repulsions, thereby attenuating the need for an energetically expensive structural change. As a result of such structural differences, the ionic transport capability was drastically reduced in both alternative functionalized MscL proteins, leading to about 5–7 permeated K^+^ ions within a 40 ns time interval, against 42 K^+^ ions for the NL system within the same time interval.

### Single-subunit engineered channel

Recent studies have demonstrated that introducing only one charge in a single subunit of the MscL hydrophobic pore (at position G22 in Eco-MscL) is sufficient to trigger channel gating in the absence of membrane tension.[16,17] Note that this is a rather different system with respect to 4L, which is also characterized by the presence of one charge in the channel core region, because in the latter case four bulky ligands prevent channel opening. Such a gating mechanism is driven by a rather peculiar phenomenon, in contrast to the charge repulsion effect described above. In fact, it has been suggested that the incorporation of one charge in the channel interior does break the short-range hydrophobic interactions by forcing hydration inside the pore.[50,51] It is reasonable to assume that already a few water molecules approaching the charged site may be enough to significantly weaken the inter-subunit interactions at the level of the most constricted channel region. Besides, experiments suggested that the partially open structure achieved via single-subunit charging is the result of an asymmetric movement of the modified subunit with respect to the others.[16,17]

**Fig 6:**
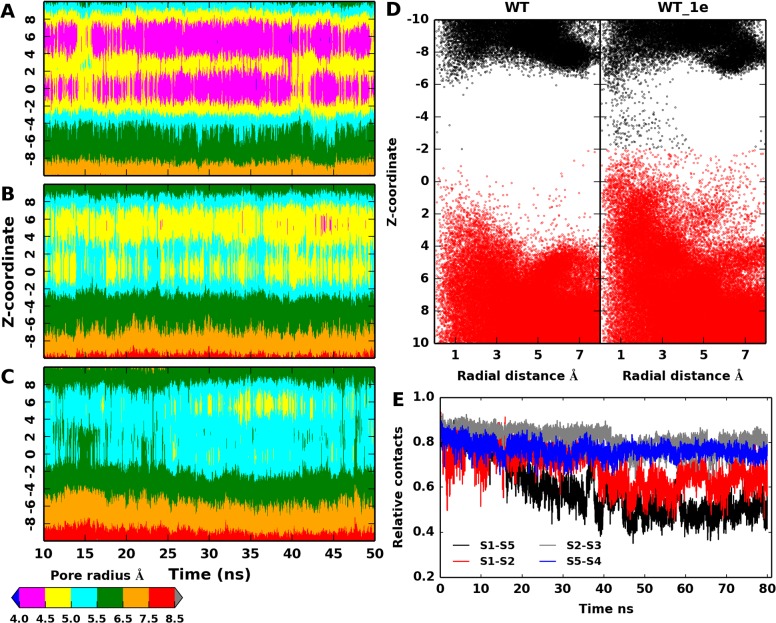
Pore radius, hydration along the channel and intersubunit contacts. Pore radius along the axial positions as a function of simulation time for (A) WT, (B) WT_1e and (C) NL models, obtained considering the backbone atoms. (D) Distribution of water molecules in the pore. For clarity, water molecules at the periplasmic vestibule of the pore are shown in black. (E) Inter-helix (TM1 vs TM1) contacts in WT_1e simulation relative to the starting configuration as a function of time.

In order to better investigate, at molecular level, the present gating mechanism, we conceived an additional model (WT_1e) in which the same negatively charged group was directly attached to the constricted site (V21) in a single subunit (subunit 1) of the WT protein, therefore mimicking the light-actuation of a single subunit of MscL. Inspection of the average pore radius along the channel axis revealed a clear expansion of the WT_1e model with respect to the WT protein (S4 Fig.), albeit to a lesser extent compared to the NL model. This can be better analyzed following the time evolution of the pore radius along the axial position, as shown in Fig. 6. In the WT simulation, the channel is constricted around the mutated site (-1< z <1) with a pore radius of < 4.5 Ang that is maintained throughout the considered time interval (Fig. 6A), while an expansion up to 5.5 Ang and 6.5 Ang is observed for the WT_1e and NL models (Fig. 6B,C). In the latter cases, a proportional expansion is observed in the regions flanking the functionalized site. The partially expanded WT_1e model was sufficient to allow ion permeation, as reported in Table 2.

Furthermore, the pore hydration upon single subunit modification was examined by monitoring the water molecules located within a defined radial distance from the channel central axis and spanning 10 Ang along the z-direction on either side of position 21. The 2D distribution of water molecules depicted as a function of axis/radial coordinates inside the pore showed a marked difference in the hydration pattern between WT and WT_1e models (Fig. 6D). In the WT model, the pore is largely hydrated at the periplasmic and cytoplasmic vestibules, while the channel core region remained occluded, as expected. This occlusion extends ~8 Ang and is consistent with previous estimates based on X-ray structure of Tb-MscL.[52] Introducing the charge in a single subunit (WT_1e) lessens the pore occlusion especially around the mutated site.

We also monitored the changes in inter-subunit contacts with time. In MscL, each TM1 helix does form contacts with other two TM1 helices from neighboring subunits. For example, residues of subunit 1 are in contact with residues belonging to subunit 2 and 5. We monitored the total contacts between the TM1 helix of subunit 1 (modified subunit) and TM1 helices of subunit 2 and 5 relative to the starting configuration (i.e., native contacts). The plot of variations in inter-helix contacts as a function of time is shown in Fig. 6E. It is apparent that between TM1 helices of subunit 1 and 2, only ~50% of the native contacts are retained in the last part of the simulation, while it is further less between subunit 1 and 5 (~40%). Intriguingly, the contacts between TM1 helices of subunit 2 and 5 with their other adjacent neighbors (TM1 helix of subunit 3 and 4, respectively) are highly maintained (~80%). These observations have shown a partial detachment of the TM1 helix of subunit 1 from its neighbors, thus supporting an asymmetric rearrangement of the pore. As a result, the WT_1e channel became permeable to ions during the simulated time interval. From the number of ions passing through the pore (Table 2), we estimated a conductance of about 80 pS, smaller than the NL model but still significant.

## Discussion

In the experimental context of ref. [15], where the light-responsive engineered MscL channel was originally presented, only binary scenarios were assumed: an “*off*”state with all subunits containing the neutral bulky photo-active ligands and an “*on*” state with all subunits converted to the corresponding charged moieties upon light irradiation. Here, taking inspiration from this pioneering study, we investigated in more detail through atomistic MD simulation the effect of charge activation. Starting from the “*off*” state (5L model), the photo-active ligands were sequentially replaced by charged groups and the corresponding functionalized MscL proteins thoroughly studied. Simulation results have shown a gradual expansion of the channel proportional to the increasing number of charges in the pore lumen (Fig. 2 and S3 Fig.), in going from the *off* (5L model) to the *on* state (NL model). In particular, the NL model displayed a conductive pore, in agreement with experiments. It is worth noting that after each chemical (electrostatic) change in the channel interior, the following structural relaxation occurred in the nanosecond timescale, thus supporting a relatively fast dynamical response of the protein channel. This result is in stark contrast to the millisecond-timescale opening observed in the native protein via the tension-induced mechanism.[53] To cope with experiments, as discussed in the following, we interpret our results as the first observation of initial MscL substates along the gating mechanism leading to the complete open channel. All systems have shown a rather stable structural character, after initial relaxation. Therefore, the observed trend in channel opening seems compliant with a gating mechanism triggered by strong charge repulsion suddenly induced in an inner region of the channel. Such an apparently simple mechanism does work so effectively only when position 21 on the TM1 helix was considered. Despite similar structural features, two alternative tested sites, namely position 17 and 25, have shown not quite the same effect, yet leading to an active MscL channel. These results are supported by several experimental evidences indicating the special role of residue G22 in Eco-MscL. Moreover, in qualitative agreement with our findings, mutagenesis studies on residues L19, V23 and G26 in Eco-MscL, which roughly correspond to L17, V21 and T25 in Tb-MscL, showed spontaneous channel activity upon charge incorporation [10,11]. In addition to such known results, in this study we attempted to provide a molecular interpretation for the effectiveness of position 21 for charge-induced activation of the MscL channel. Indeed, it has been found that this site is the most “sensitive” for the reason that it is, at the same time, the most constricted within the pore hydrophobic stretch and the less exposed to solvent. Accordingly, mutagenic studies have shown that V21 of Tb-MscL participate in the lock and reported that V21D mutant decreased the energy barrier for gating, resulting in gain-of-function phenotype.[54,55]

On the other hand, inspired by recent experiments that demonstrated channel activation upon chemical modification of a single subunit of Eco-MscL,[16,17] an additional model was considered where the charged group was introduced at only one site (i.e. V21 of subunit 1) of the Tb-MscL protein. Our simulation revealed a partially expanded pore allowing ion translocation (Fig. 6B, Table 2), again in qualitative agreement with experiments. In contrast with the previously described charge-repulsion trigger, the channel activation is achieved through a forced hydration into the channel core region, thus leading to pore expansion. The present gating mechanism is consistent with the proposed hydrophobic breaking effect[1] as observed with single-charge subunit substitution[16,17] or with polar residue substitution.[9] In fact, it has been observed that replacement of hydrophobic residues with hydrophilic ones triggered channel gating at reduced or no applied tension. Hence, it has been suggested that hydrophobic residues in the narrow pore region do create a functional channel block, which can be easily destabilized upon incorporation of polar (charged) residues, resulting in a conductive channel configuration through structural rearrangements.[50,51]

Our investigation has also unraveled further molecular details concerning the asymmetric motion of the only charged subunit with respect to the others. Such a structural transition, not triggered by a strong repulsive interactions, is possibly another confirm of the highly flexible character of the MscL channel. This is perhaps not surprising if we consider that the natural activation mechanism of the mechanosensitive channels does require a considerable structural change driven solely by membrane tension, whereas other protein channels activated by different stimuli (e.g., voltage and ligands) are significantly more rigid and insensitive to membrane stress. Furthermore, the MscL transition from the closed to the open state has been described as an iris-like symmetric motion of all subunits.[56–59] However, recent evidences indicated that MscL gating could also follow an asymmetric route.[60,61] In our model (WT_1e), the charge introduced in a single subunit resulted in a gradual weakening of the contacts between the latter subunit and the other pore-forming helices, while the contacts between the other native subunits have been consistently maintained (Fig. 6E). These findings supported a progressive and asymmetric detachment of one subunit with respect to the others, an observation in agreement with experiments pointing towards a nonlinear contribution of individual subunits to the tension sensitivity.[16,17]

While the present computational investigation has provided an overall consistent picture with respect to known experiments, especially for the WT_1e and NL models representing activated MscL protein channels, some important details contrast with experimental findings. In particular, the extent of pore expansion achieved in our simulations is significantly smaller than experimental predictions. The introduction of a single charge on a pore residue in one subunit of Eco-MscL has been estimated to lead to a pore diameter of up to ~10 Ang.[17] In our work, even for the most expanded NL model, we obtained a pore diameter of about 5.5 Ang around the functionalized site (including side chains). As a result, the computed conductance for both WT_1e and NL systems was found to be underestimated by a factor of about ~3 with respect to the experimental counterpart (e.g, NL model provided a conductive channel of 140 pS instead of ~500 pS[15]). We note, however, that a discrepancy between theory and experiments of the same order is not unusual,[62,63] as probably due to other important factors usually difficult to include in the molecular modeling of ion conductance, such as a more complex lipid composition, membrane pressure fluctuations, polarization effects, and so on. As an example, in a previous computational study the impact of different lipids on MscL has been addressed:[23] therein, some important structural rearrangements of the protein have been noticed, especially in the C-terminal region, though the MscL pore has resulted unaffected. In addition, a recent study showed that a specific lipid composition, including phosphatidylinositol, is crucial for allowing a physiological opening of Tb-MscL.[64]

In our case, we envisage that the expanded pore likely represents a sub-conducting state of the channel. MscL has been shown to visit sequential intermediate states during the transition from the closed to the open state. The charge-induced activation apparently populates the same initial substates achieved by the natural trigger, i.e. membrane tension.[17] Accordingly, our models presumably captured such initial substates visited upon activation whose diameter has been previously estimated to lie in the 5–10 Ang range (the observed stability of the NL model channel at high temperature confirmed such a substate character). In other simulation studies focused on the natural MscL gating mechanism, a membrane tension or radial forces on protein were used to achieve an extended opening in an affordable time.[18,56,65–67] Here, we aimed at addressing the charge-triggered structural events in the absence of tension, therefore no external forces were applied to the protein or the membrane. In addition, other possible causes for the observed disagreement may be related with protein structural differences between the Eco and Tb-MscL. Indeed, Tb-MscL has been shown to gate at about twice the tension needed to gate Eco-MscL, when expressed in *E*.*coli* spheroplasts (*in-vivo)* or reconstituted in azolectin liposomes (*in-vitro*), a feature that was observed when the crystals of Tb-MscL was resolubilized and functionally reconstituted.[55] Hence, despite the high conservation of the MscL pore residues among different organisms,[54] structural features of individual MscL may have implications on its sensitivity to perturbations and interactions with the lipid environment.

## Conclusions

The bacterial mechanosensitive channel of large conductance (MscL) has been conceived as an ideal activated nanovalve for controlled drug delivery with several advantages that include: i) large conductance, ii) non-selective pore, iii) easy isolation from cells, iv) in-vitro translation, v) reconstitution in synthetic lipids and vi) charge-induced activation of channel gating. In particular, incorporation of photo-active groups into the hydrophobic channel core through site-specific mutations and chemical synthesis has enabled a light-activated gating mechanism, which offers a high spatio-temporal control without affecting the environment. In addition to these relevant features, the present computational study has unraveled a few interesting details concerning the MscL charge-induced gating: (1) a fast pore-opening dynamics (nanosecond timescale) leading to initial conducting substates, (2) a high sensitivity of the channel structural expansion to several factors (including the choice of the functionalization site in the channel interior, the number of incorporated charges, the ion distribution along the pore) and (3) an asymmetric subunit movement upon single-charge incorporation into the pore. We note that the observed sensitivity of charge perturbations towards selected channel interior sites may support the need for a preliminary molecular modeling of the detailed triggering events leading to pore gating. As well, we note that independent subunit movements upon charge activation not only support the proposed asymmetric motion in MscL channel opening but also do suggest the possibility of a subunit-specific triggering to achieve pores of desired sizes.

## Supporting Information

S1 Fig Root mean square deviations as a function of time. Backbone RMSD, calculated with respect to the starting configuration of respective simulations. The right panel represents the distribution of the RMSD values over the last 20 ns.
(TIF)

S2 Fig Per residue averaged root mean square fluctuations. Backbone RMSF mapped onto the average structure. The protein is represented as ribbon whose color (green to red) and thickness is proportional to the RMSF values.
(TIF)

S3 Fig Distribution of the pore area per residue. Pore area estimated for a stretch of residues lining the TM1 helix for WT (gray), 5L (black) and NL (magenta). The vertical dashed line corresponds to the X-ray structure.
(TIF)

S4 Fig Average pore radius as a function of axial position. Average pore radius along the axial positions, obtained considering the backbone atoms.
(TIF)

S1 Table Backbone RMSD (in Ang) of the engineered MscL channels. (DOC)
