# Supplementary figures and images for: Breaking the Hydrophobicity of the MscL Pore: Insights into a Charge-Induced Gating Mechanism
Source: PLoS One. 2015 Mar 31;10(3):e0120196. doi: 10.1371/journal.pone.0120196 (PMC4380313; doi:10.1371/journal.pone.0120196)

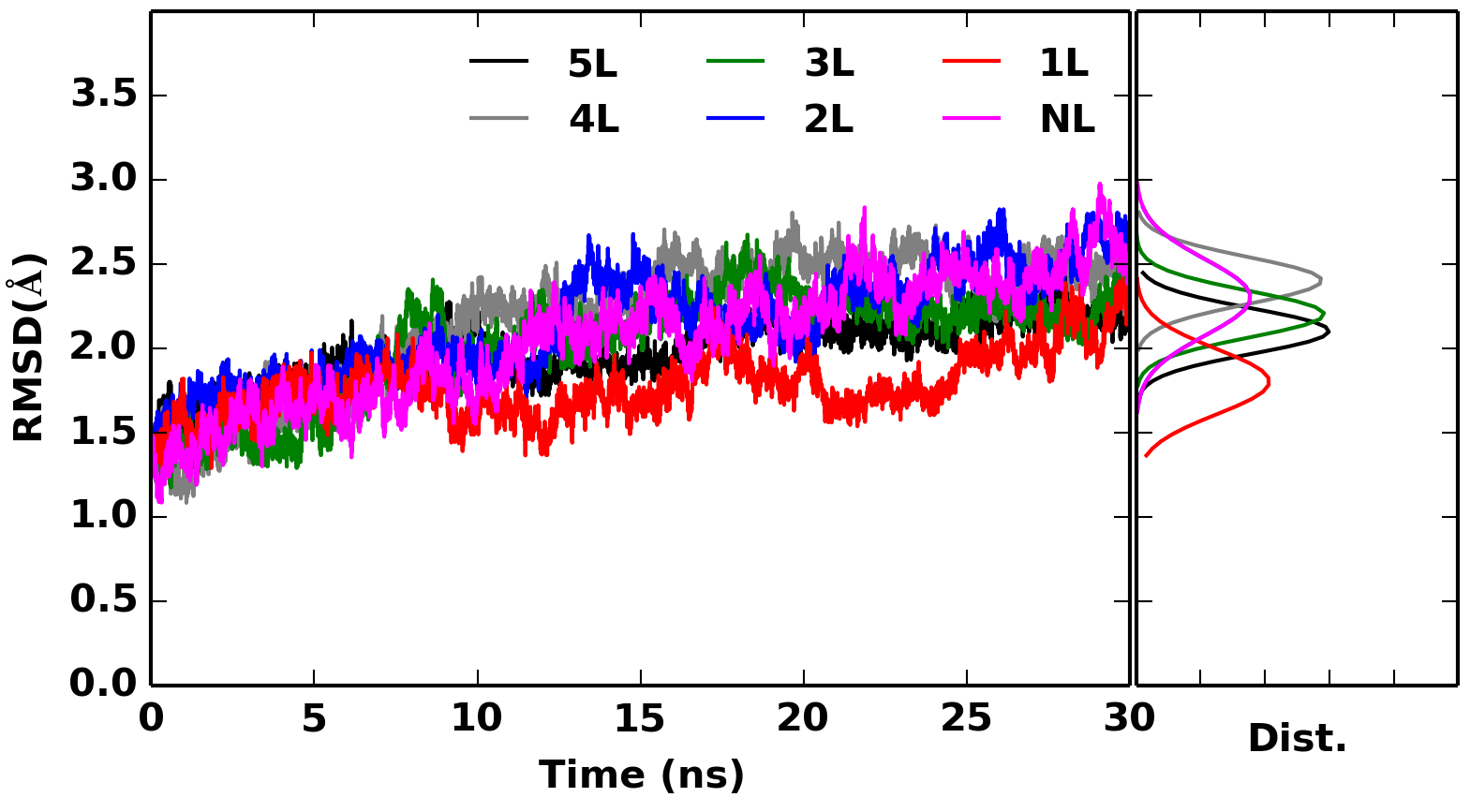

Supplement: S1 Fig — Backbone RMSD, calculated with respect to the starting configuration of respective simulations. The right panel represents the distribution of the RMSD values over the last 20 ns. (TIF) [file pone.0120196.s001.tif]

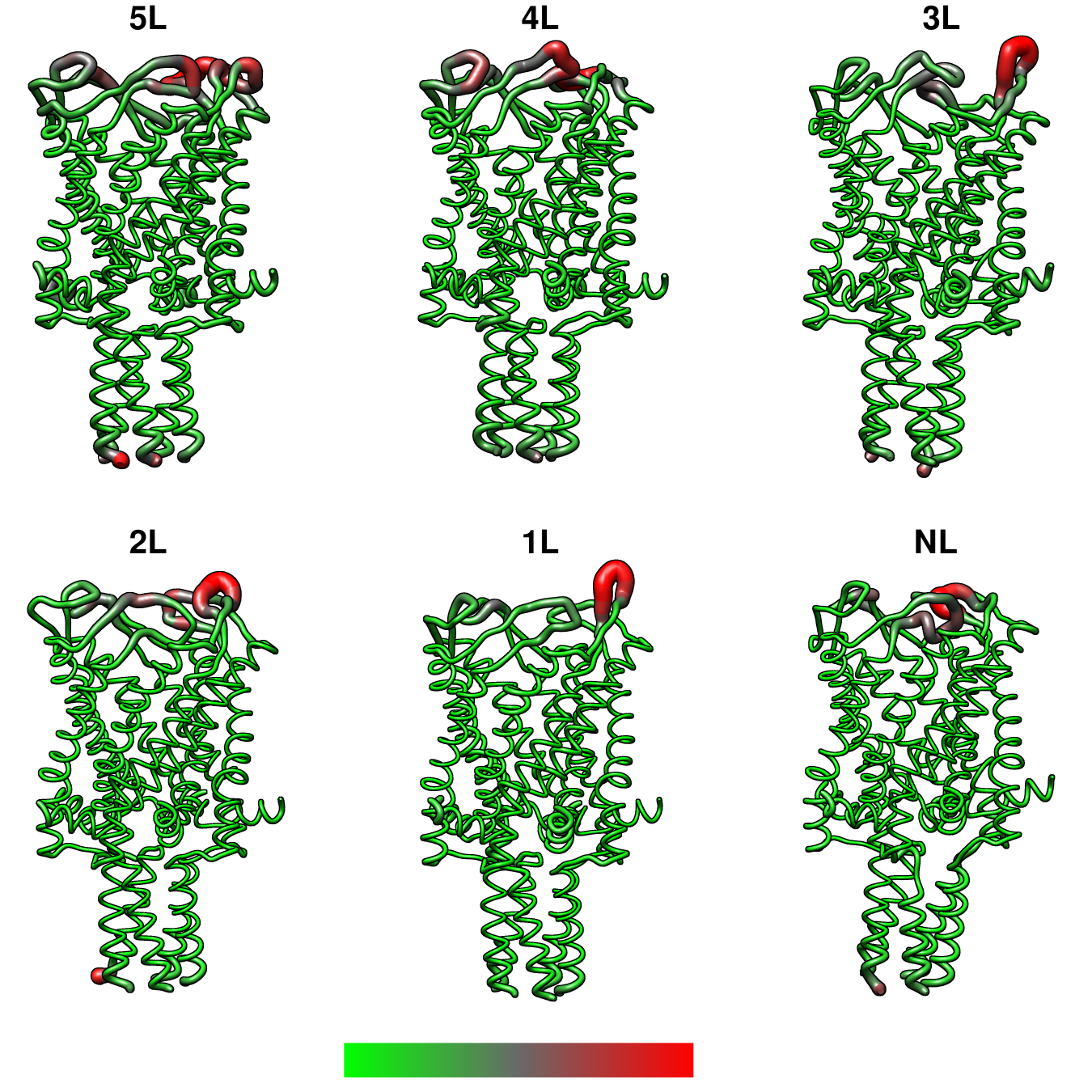

Supplement: S2 Fig — Backbone RMSF mapped onto the average structure. The protein is represented as ribbon whose color (green to red) and thickness is proportional to the RMSF values. (TIF) [file pone.0120196.s002.tif]

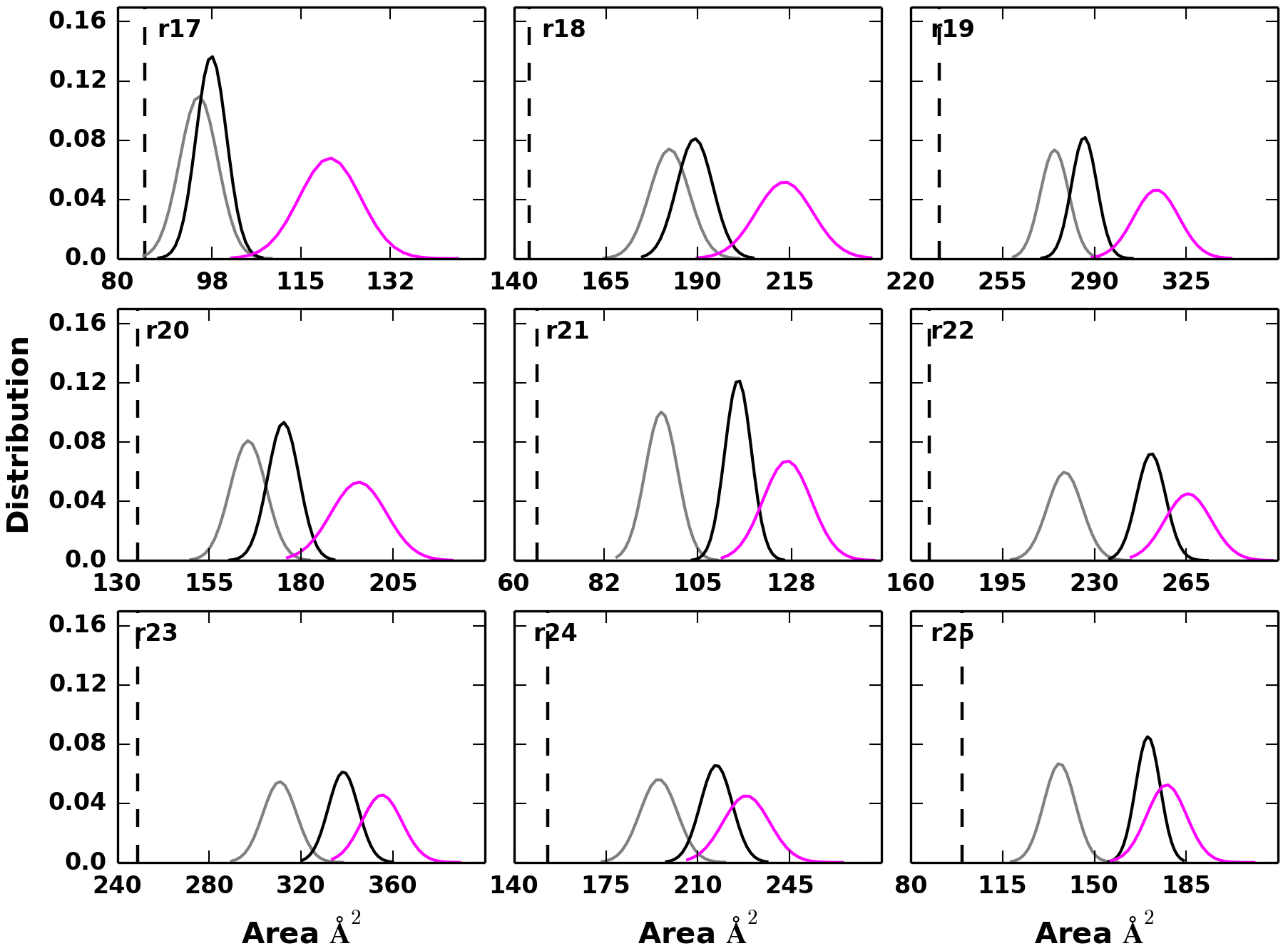

Supplement: S3 Fig — Pore area estimated for a stretch of residues lining the TM1 helix for WT (gray), 5L (black) and NL (magenta). The vertical dashed line corresponds to the X-ray structure. (TIF) [file pone.0120196.s003.tif]

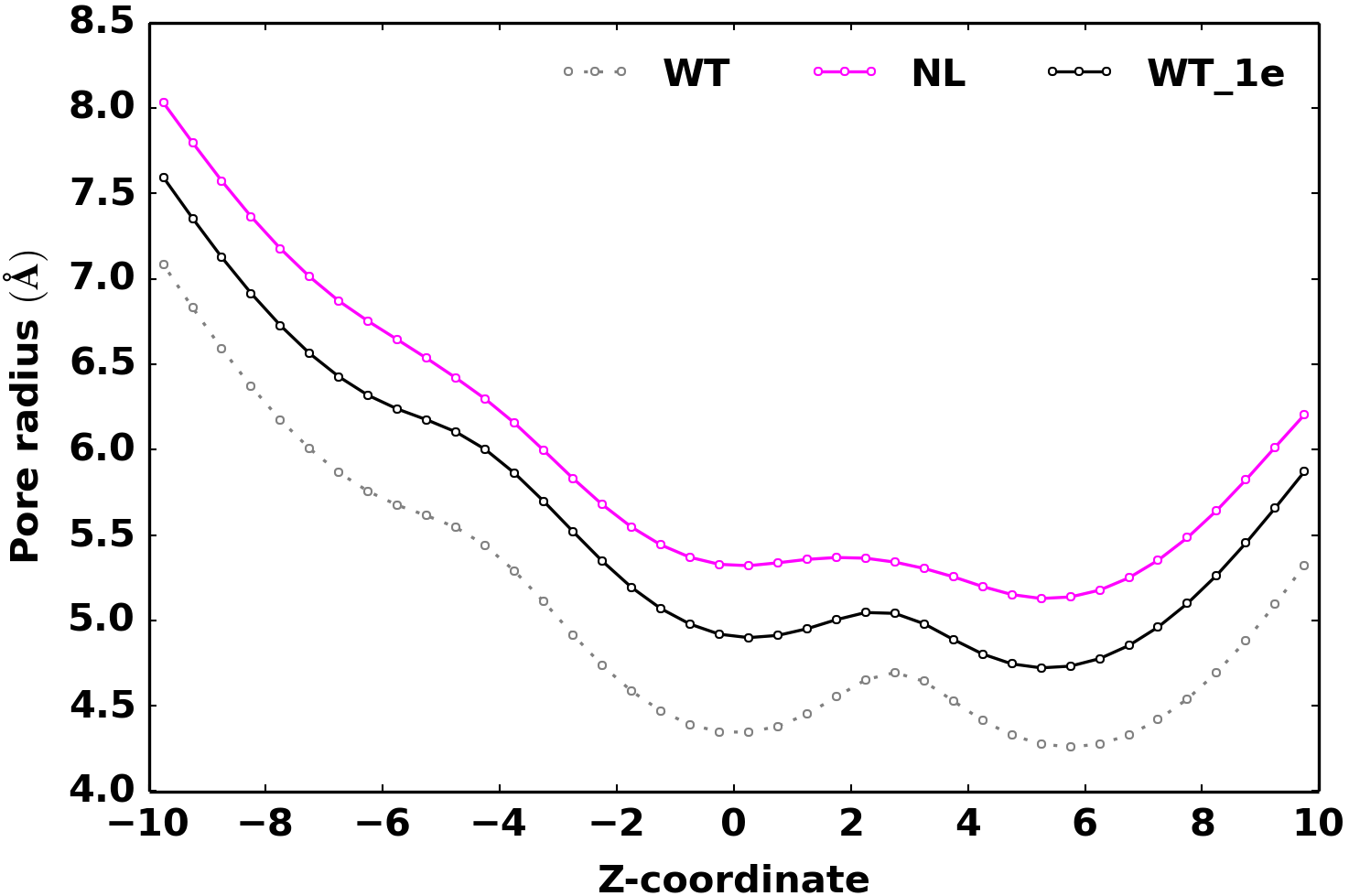

Supplement: S4 Fig — Average pore radius along the axial positions, obtained considering the backbone atoms. (TIF) [file pone.0120196.s004.tif]
