# Supplementary material for: Breaking the Hydrophobicity of the MscL Pore: Insights into a Charge-Induced Gating Mechanism
Source: PLoS One. 2015 Mar 31;10(3):e0120196. doi: 10.1371/journal.pone.0120196 (PMC4380313; doi:10.1371/journal.pone.0120196)
Supplement: S1 Table — (DOC) [file pone.0120196.s005.doc]

**Table S1. Backbone RMSD (in Ang) of the engineered MscL channels**

| Model | A | B | C | D |
| --- | --- | --- | --- | --- |
| 5L | 2.1 (0.13) | 2.4 (0.08) | 1.6 (0.10) | 1.0 (0.11) |
| 4L | 2.4 (0.14) | 2.9 (0.12) | 1.7 (0.08) | 0.9 (0.11) |
| 3L | 2.2 (0.14) | 2.9 (0.12) | 1.8 (0.10) | 0.9 (0.12) |
| 2L | 2.3 (0.22) | 3.2 (0.18) | 1.8 (0.10) | 1.0 (0.17) |
| 1L | 1.9 (0.20) | 2.9 (0.18) | 1.9 (0.08) | 0.9 (0.12) |
| NL | 2.3 (0.22) | 3.4 (0.17) | 1.8 (0.08) | 1.5 (0.13) |

RMSD evaluated with respect to (A) the starting configurations and the X-ray structure for (B) whole protein, (C) transmembrane and (D) C-terminal domains. Standard deviations are in parenthesis.
